# The reporting requirements of case reports and adherence of case report reporting guidelines in medical journals: an analysis of the authors’ guide sections

**DOI:** 10.1186/s13256-022-03710-2

**Published:** 2023-01-05

**Authors:** Abolfazl Taheri, Peyman Adibi, Mojtaba Sabbagh Jafari, Marzieh Saeedizadeh, Alireza Rahimi, Alireza Abbasi

**Affiliations:** 1 Department of Medical Library and Information Sciences, School of Management and Medical Information Sciences, Isfahan University of Medical Sciences, Isfahan, Iran; 2 Integrative Functional Gastroenterology Research Center, Isfahan University of Medical Sciences, Isfahan, Iran; 3 School of Computer Engineering, Vali -e- Asr University of Rafsanjan, Rafsanjan, Kerman Iran; 4 Knowledge and Information Science, Public Libraries Foundation, Tehran, Iran; 5 Clinical Informationist Research Group, Health Information Technology Research Center, Isfahan University of Medical Sciences, Isfahan, Iran; 6 School of Engineering and Information Technology, University of New South Wales, Canberra, Australia

**Keywords:** Reporting guideline, CARE guideline, Case report, Medical journalism, Authors’ guide, Reporting requirements

## Abstract

**Background:**

Owing to the growth of case reports and changes in the policy of journals in publishing this evidence, the need to standardize them is felt more than before. Therefore, in this study, the authors’ guide of medical journals indexed in the Scopus database that published most of the case reports has been analyzed to identify the reporting requirements and emerging case report types.

**Methods:**

A total of 50 journals were selected from the Scopus citation database (the world’s largest knowledge base) that published most of the case reports. These and the authors’ guideline section on the types and requirements of writing case reports were analyzed by inductive content analysis.

**Results:**

Most of the case reports were published in the fields of dermatology and surgery and general medicine. Reporting requirements in author’s guide are grouped in four categories: (1) reasons for publication or content value, (2) emphasis on the patient consent form and confidentiality, (3) emphasizing the constraints on the word count and limitation, and (4) recommendation for structure and reporting elements. In terms of adherence to the reporting guidelines, 76% of journals do not adhere to any reporting guideline. In addition, 13 types of case reports were identified in these journals, among which traditional case reports, clinical image, letters, and case series were the most widely used formats.

**Conclusions:**

Improving the publication processes of case reports has been left unattended by international organizations. The policies of journals need to become more integrated, and reporting guidelines should be modified or redeveloped to enhance the quality of publications, cover different reporting requirements, and consequently, benefit from the evidence value available in case reports.

**Supplementary Information:**

The online version contains supplementary material available at 10.1186/s13256-022-03710-2.

## Introduction

The number of published clinical case reports (CCRs) has increased considerably over the past few years. Statistics show that almost half of the articles in this area have been published during the past 20 years [1]. Although several credible medical journals have abstained from publishing such articles due to issues concerning scientometrics and the absence of statistical methods [2, 3], many other journals keep creatively publishing them in different formats [4].

The huge number of published CCRs makes it necessary to control the quality of such clinical evidence. The only serious undertaking so far is the development of a reporting guideline called CARE CAse REport guidelines (CARE) [5]. However, it is unknown what percentage of journals have adopted these guidelines [6].

CARE includes only reporting items, and no integrated policies and guidelines have been introduced by international organizations concerning the publication of CCRs. Thus, as the authors’ guidelines are the first and the only way of communication with authors in the journals that publish CCRs, they should include accurate and clear instructions on the reporting details and requirements of such studies. Therefore, the first step toward standardizing and proposing international criteria for CCRs is the investigation of reporting requirements for the journals that publish the most number of CCRs.

In view of the above, the present study aimed to pursue the following secondary objectives:

1. Identify the reporting requirements and considerations presented in the “Authors’ guide” sections of the journals publishing most of case reports.
2. Determine the degree of adherence to the CARE guidelines in the journals publishing most of case reports.
3. Characterize the typology of the case reports published in the journals publishing most of case reports.

## Materials and methods

### Study design

The current study applies a qualitative approach and an inductive content analysis (qualitative and quantitative) technique. Indeed, content analysis was applied as a way to investigate the “Author’s guide” sections of the English medical journals with the largest number of published CCRs. The investigations were conducted during February–March 2021. We used the consolidated criteria for reporting qualitative research (COREQ) as the reporting framework for this study [7].

### Study population

As the process of investigating all journals was lengthy and the study was qualitative in nature, the authors decided to investigate the sample of journals. Thus, purposive sampling was used to achieve an eligible sample among journals. This method involves identifying and selecting samples of communities that are especially knowledgeable about or experienced in a phenomenon of interest [8]. Among the purposeful sampling strategies in Palinkas *et al*., Criterion-i sampling (criterion of inclusion in a certain category) was chosen as a purposeful sampling strategy [9]. In this study, inclusion (or eligibility) criteria included: (a) journals indexed in SCOPUS; (b) English language journals; and (c) journals rated as top journals with the highest number of published case reports.

To identify the top journals that have published the highest number of case reports since 2010–2021 in SCOPUS, the following search strategy was applied.

**Table:** 

Search strategy
(TITLE (“case report*”) OR TITLE (“case series”) OR KEY (“case report”))
AND (LIMIT-TO (SRCTYPE, “j”))
AND (LIMIT-TO (SUBJAREA, “MEDI”) OR LIMIT-TO (SUBJAREA, “BIOC”) OR LIMIT-TO (SUBJAREA, “IMMU”) OR LIMIT-TO (SUBJAREA, “NURS”) OR LIMIT-TO (SUBJAREA, “PHAR”) OR LIMIT-TO (SUBJAREA, “DENT”) OR LIMIT-TO (SUBJAREA, “HEAL”))
AND (LIMIT-TO (LANGUAGE, “English”))
AND (LIMIT-TO ( PUBYEAR, 2010–2021))

A comma-separated values (CSV) file of the results was obtained, and the journals with the maximum number of published case reports were selected from the “Source Title” column. As the process of investigating all journals was lengthy and the study was qualitative in nature, the authors decided to investigate the selected journals. This is because it was expected that the journals with the largest number of published reports had more accurate and enhanced “Authors’ guide” sections in terms of the reporting requirements of case reports. Thus, 50 journals that published the most number of case reports were selected as the population of this study for further analysis.

It should be noted that the journals that dedicatedly published CCRs (that is, had “case report” in their titles), the journals that did not publish CCRs anymore, and the journals with unavailable or inactive websites were excluded, and the population was formed merely out of the journals that published CCRs in addition to other formats of studies. We distinguished these general journals from dedicated case report journals. In general journals, only a part of the authors’ guide has addressed the requirements of the journal for publishing a case report as one of the types of articles in that journal, while in dedicated CCR journals all the authors’ guides section are devoted mainly to discussing the requirements for publishing various types of CCRs in those journals.

### Analysis: the steps and units

**Fig. 1:**
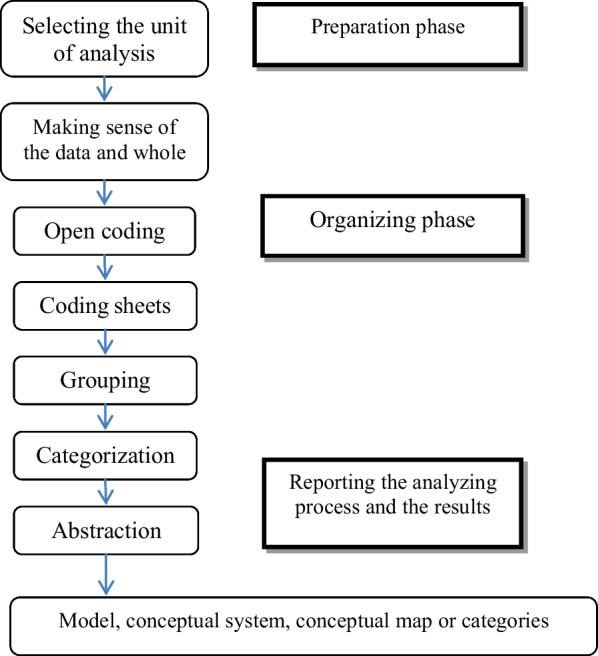
Inductive classification scheme in the content analysis process (according to Elo & Kyngäs [10])

Two team members, experienced in qualitative research methods, guided the analytic process using inductive content analysis. The organization of the qualitative data in the inductive content analysis includes open coding, creating categories, and abstraction [7]. Figure 1 illustrates the general scheme and stages of the content analysis implemented in the this study.

The whole content dealing with the discussions of reporting a case report presented in the “Authors’ guide” sections of a journal was considered the unit of analysis, which includes texts, images, tables, templates, and PDF files in the “Authors’ guide” sections. In addition, two sample case reports were investigated from each journal.

The content analysis began with the repeated reading of the “Authors’ guide” sections to find a general feeling and make sense of the data. In the stage of open coding, the textual data were inserted into Citavi, while quantitative data such as frequency and percentage were entered into Microsoft Excel. The sets of codes entered to Citavi were classified according to their similarities or differences, and they were later subdivided after being coded. The classification procedure was conducted to decrease the number of classes. This stage aimed to provide a method of describing the phenomena and increase the understanding and production of knowledge [7]. More specific labels were applied to group the data. The classes were named, and the subclasses were categorized in similar forms. Then, the themes were identified. Abstraction refers to the creation of a general description of a research topic.

### Reliability of the content analysis

The measures of credibility, transferability, dependability, and confirmability were applied to investigate the reliability of the data. To achieve credibility, the format and structure of two recently published CCRs in each journal were investigated in addition to the text of the “Authors’ guide” sections. This was conducted so that important pieces of data may not remain unclassified or ignored. Concerning transferability, as the researcher in qualitative studies is supposed to present the set of data and textual descriptions completely and richly, reporting the findings thoroughly and stating and estimating them conveniently was attempted. The two researchers reviewed the stages of data collection and analysis and approved the results to attain dependability. Moreover, confirmability was investigated by meticulous and repeated reviews and the researchers’ long-term involvement with the data, analyses, and results.

## Result

**Fig. 2:**
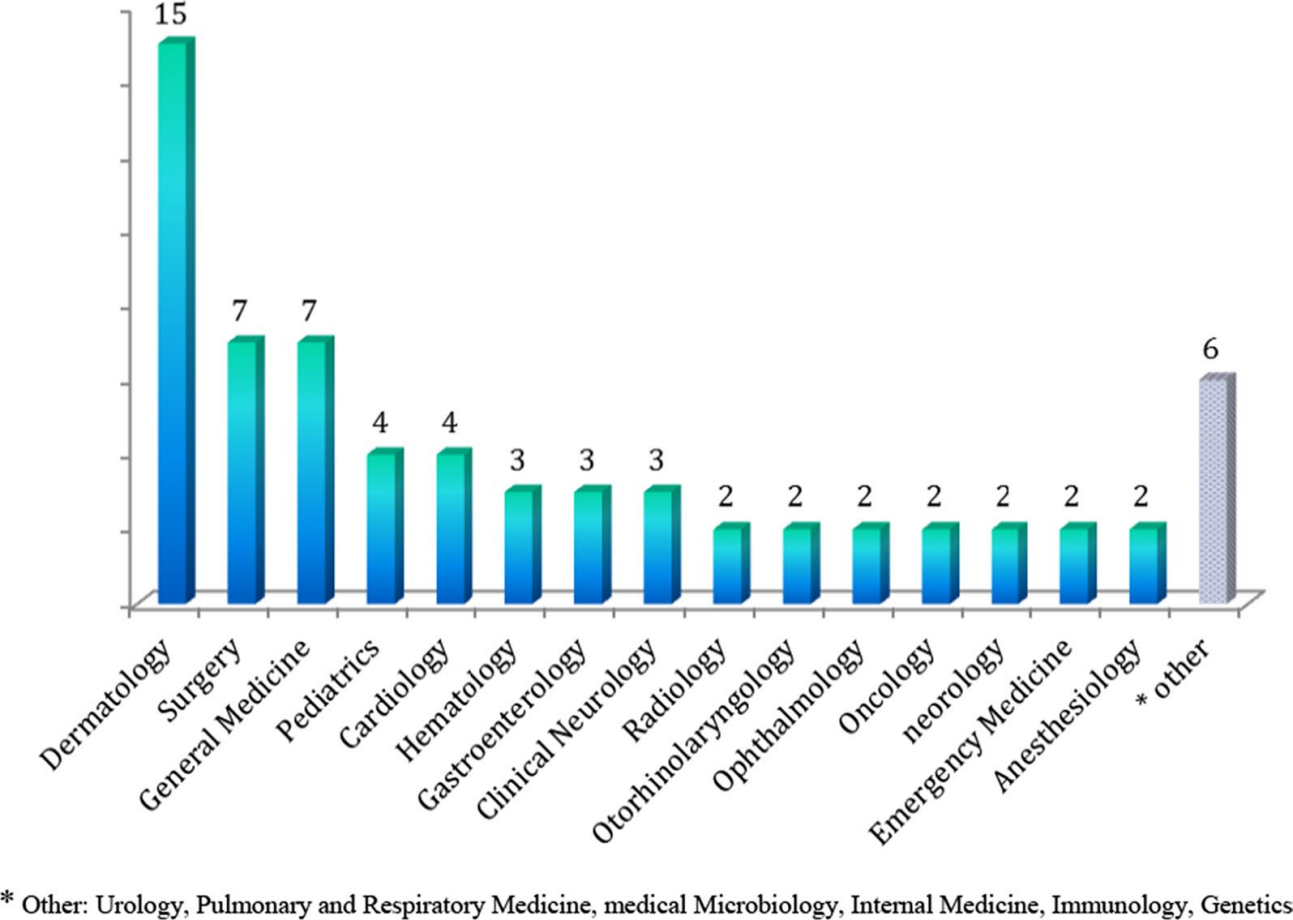
Frequency distribution of 50 medical journals publishing most of CCRs by subject. *Other: urology, pulmonary and respiratory medicine, medical microbiology, internal medicine, immunology, genetics

Out of the 50 studied journals, 15 journals (30%) were in the field of dermatology. Moreover, surgery and medicine (7 journals) ranked next. The lower frequencies of the other areas are illustrated in Fig. 2.

### Objective 1: identify the reporting requirements and considerations presented in the “Authors’ guide” sections of the journals publishing most of case reports

The analyses conducted to identify the reporting requirements and considerations of the CCRs indicated that most of the content can be classified as: (a) reasons for publication or value of the content, (b) emphasizing the patient consent form and confidentiality, (c) emphasizing the constraints on word count and limitation, and (d) recommendation for the structure and reporting elements. The first category reflects the characteristics and values of submitted case reports that should meet the publication criteria of the target journal.The second category contains recommendations for obtaining consent forms and keeping patient de-identifiable. Moreover, the third category indicates the policies of the journals regarding the limitation of words and tables and the whole manuscript. Finally, the fourth category included items and sections that have to be adhered to in the case report manuscript. Each requirement will be discussed below.

#### Reasons to publish/the value of content

A total of 42% of the journals (21 journals) discussed the content value of the CCRs in their “Authors’ guide” sections. Thus, the most considerable reasons to publish included the following: rarity, unusual or unique aspects (not previously published), atypical evolution/pathogenesis, therapeutic and diagnostic innovations, emerging disease, unexpected outcome, or unusual therapeutic adverse events.

The “Authors’ guide sections of journals such as the *American Journal of Emergency Medicine *explicitly indicated that the treatment process of the clinical cases should be completed, in addition to the other characteristics of the report such as being unique and new. In addition, the *American Journal of Medical Genetics*emphasized that CCRs should be representative of diverse populations. “Educational value” was another significant feature that the investigated journals considered as acceptance criteria of CCRs. In other words, the received case report must be attractive for the journal’s readers and provide an educational message. In this regard, various journals publish CCRs in educational formats such as quizzes and continuing professional development (CPD). The format of these CCRs is structured so that the main focus is not on the uniqueness of the clinical case, but the educational message should be framed in different forms for the audiences.

Only the *British Journal of Dermatology*prioritized the publication of case reports that contained a new research hypothesis. In addition, the journals regarded the evidence value of literature review in case reports contradictorily. For example, the “Authors’ guide” sections of the *Child’s Nervous System* journal indicates that the received case report can include the review of the related literature, while *The Annals of Thoracic Surgery* prohibits this section.

#### Emphasis on patient consent form and confidentiality

In 70% of the journals, it was explicitly indicated that the publication of the case report depends on providing informed consent statements and obtaining them from patients or their relatives. The “Authors’ guide” section of the *Ear, Nose, and Throat Journal* indicates that authors should only state in the “Informed Consent Statement” that the consent form was obtained, and there is no need to upload any forms in the journal management system. The statement was defined as an essential component of introduction in the *Journal of Clinical Anesthesia*. The “Authors’ guide” section of the *Brasileiros de Dermatologia* emphasized that the patients’ data should not be changed to maintain their privacy, and only the identifiable details should be omitted. In addition, information such as the name of patient and hospital and the exact time should be removed from images, and the patients’ consent for the publication of images should also be obtained (*Pan African Medical Journal*). Moreover, the inclusion of demographic information should correspond to the topic and clinical context and should not lead to the identification of a particular patient (*Clinical and Experimental Dermatology*). In addition, the person who consented should be determined. The “Authors’ guide” section of *Child’s Nervous System* indicates that obtaining ethical approval is necessary to publish case reports, and authors should obtain it in addition to the patients’ consent forms from the institute where they worked.

#### Considering the constraints on word count and limitation

The findings indicated that only 10 journals had not set any word count constraints in the CCRs. In other words, 80% had set at least one form of limitation (the number of words in the whole text, authors, abstract, figures and images, and references) for CCRs. Moreover, 35 journals (70%) had set some constraints concerning the number of words for the whole text.

The “Authors’ guide” sections mostly deal with the word count constraints in the whole text or abstract, the number of images and tables, the number of authors, and references. This approach mostly originated from the traditional policies of print publication. Thus, journals tend to publish CCRs with a minimum number of words. Concerning the constraints as to the number of authors, it should be stated that CCRs involve less complexity in comparison to original or review articles, and the number of authors in such articles is expected to be less than others. Thus, the number of authors in CCRs can be four to seven, depending on the policies of each journal.

#### Recommendations for the structure and reporting elements

In addition to items related to writing limitation, 22 of the “Authors’ guide” sections (44%) included recommendations as to the reporting sections and elements of CCRs. Out of the 22 journals, only 4 journals had presented a reporting template as a Microsoft Word file for CCRs. These journals included *Indian Journal of Ophthalmology*, *Pan African Medical Journal*, *American Journal of Medicine*, and *Dermatology Online Journal*.

The general structure of a case report according to the investigated journals included the following sections: title page (including the title), authors’ affiliation, funding information, abstract, keywords, introduction, case presentation, discussion, conclusion, acknowledgments, conflict of interest, authors’ contribution, references, images and tables (accompanied with textual description, caption, or legend), and supplemental data (for example, chromosome data, large tables, additional photos). To increase the educational value of a case report, journals such as *QJM* required the authors to provide “Learning Points for Clinicians” in the discussion section, preferably in bullet format. In this regard, authors were required by the *British Journal of Dermatology* to state learning points in a 70-word section titled “bulleted statements” and answer the following two questions: “What is already known about this topic?” and “What does this study add?”

A similar reporting item was regarded as essential by the *American Journal of Emergency Medicine*, as the “Authors’ guide” section indicates the submitted CCRs should have a section titled “Why should an emergency physician be aware of this?”

One of the most unique reporting items of CCRs is concerned with the requirement of the *Pediatric Dermatology Journal*. In the guide it was stated that “firstness claims” in the CCRs should be based on a detailed search methodology. The explanations in this regard were supposed to be submitted to the editor as a letter. The “firstness statement” required the authors to claim that their report includes the first clinical case occurring across the world or in a particular clinical context.

### Objective 2: determine the degree of adherence to the CARE guidelines in the journals publishing most of CCRs

**Fig. 3:**
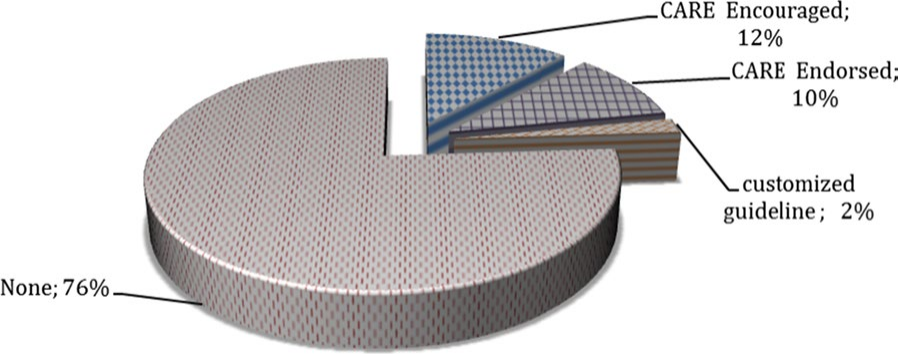
Degree of adherence to CCR reporting guidelines in the medical journals publishing most of the CCRs (with focus on CARE guidelines)

To fulfill the second objective, the “Authors’ guide” sections were analyzed to identify different types of reporting guidelines that were recommended or required by the journals. As the researchers were familiar with the process of writing and publishing CCRs and their reporting guidelines, this section of the study focused on the CARE guidelines. Figure 3 indicates the adherence of each journal to CARE guidelines.

In this study, the term “encouraged” means that the journal only mentioned the existence of the CARE guide and did not require the authors to adapt to its items. On the other hand, “endorsed” means that the journal required adherence to the guideline, and the authors had to submit their CCRs according to its reporting descriptions.

The findings indicated that 76% of the journals did not present any guidelines for reporting the CCRs (shown in Fig. 3). The “Authors’ guide” section of 6 journals (12%) indicate that the authors can adapt their reports to the CARE guide in a voluntary/encouraged way to increase the quality of their paper. Out of the investigated 50 journals, only 5 instances (10%) endorsed CARE. Moreover, the *Journal of Contact Dermatitis* developed a customized guideline titled the “Contact Allergy Case Reports Guidelines”.

### Objective 3: characterize the typology of the CCRs published in the journals publishing most of the CCRs

As it can be observed in Table 1, 13 types or formats of CCRs were identified in the journals. Apart from the classic CCRs that were published in 36 journals, the format of “clinical image” was found to be the most popular (in 25 journals) among the published CCRs. Other popular formats included “letter” and “case series.” A brief description concerning the goal and structure of the identified formats is presented in Additional file 1: Appendix S1.

**Table 1:** Typology of CCRs published in medical journals (*N* = 50)

#	Category	Subcategory	No. of journals/journal name
1	Case report	Traditional case report	36
		Case-based review	Child S Nervous System
		Clinical/Technical note	Pediatrics International; Neurology
2	Case series	–	11
3	Brief report	–	5
4	Video	–	5
5	Letter to editor	–	18
6	Round	–	International Journal of Dermatology; Urology
7	Brief/short communication	–	Child S Nervous System; Journal of Clinical and Diagnostic Research
8	Clinical problem solving	–	New England Journal of Medicine
9	CPD	Clinicopathological Cases, Therapeutic Vignette, Genetic Report, A Memorable Patient, Patient Viewpoint	Clinical and Experimental Dermatology
10	Case conferences	–	Journal of Cardiothoracic And Vascular Anesthesia
11	Clinical image	–	25
12	Diagnostic dilemmas	–	Journal of Cardiothoracic and Vascular Anesthesia
13	Quiz	Challenge	6
		Practical teaching case	Gastroenterology
		Clinical quiz	9
		Brief case	Journal of Clinical Microbiology

*CPD* Continuous professional development

## Discussion

Concerning the developments of scientific publications and the involvement of journals with “scientometrics,” creditable medical journals have decreased the acceptance of case reports due to the reduction of impact factor (IF) [2, 3]. However, the journals that intend to publish these valuable clinical articles have managed to do that by making changes in publication policies and introducing new formats. The typology identified in this study showed that there are at least 13 types of case reports. These typologies intend to overcome the issues of scientometrics and attract more audiences [4, 11]. Some of these formats have become educational tools for fellowship courses and novice clinicians [12]. Parker *et al*. [13] believe that educational formats such as clinical challenges enable students to become familiar with different clinical scenarios and enhance their critical thinking skills.

Another important note to bear in mind is that the “Authors’ guide” sections pay considerable attention to obtaining patients’ consent forms. Though such forms act as the key to the ethical publication of case reports and are one of the main publication components in the journals, only 70% of the journals require the researchers to obtain an informed consent form from the patients or their relatives. If journals do not require this, the principles of privacy and confidentiality that are among the most fundamental ethical principles of modern medicine may be breached. Compared with the findings of Yoshida *et al*. [14] that indicated obtaining a consent form is required in only 40% of 491 academic journals, the results of the current study pointed to increased awareness among the journals. Thus, the organizations involved in medical ethics should endeavor to publish a global consent form for case reports and oblige journals to adhere to this form and get the patient’s consent for the publication of CCRs. It is recommended that journals should carefully define the ethical considerations involved in the process of publishing CCRs (e.g., plagiarism issues, data availability, and ethics committee approval) in their “Authors’ guide” sections.

Though it was expected that the “Authors’ guide” sections of the journals with the maximum number of published CCRs should contain detailed reporting requirements, no accurate and structured recommendation was found concerning the reporting requirements of such evidence. Thus, the “Authors’ guide” sections mostly discussed the constraints on word count and the volume of content. This was in line with the findings of Sorinola *et al*. [15], where it was argued that a standard “Authors’ guide” section should be designed specifically for CCRs. In addition, for the writing of an editorial article, Gupta and Gaba [16] stated that whenever possible, use short and concise sentences, edit your own work, and dispose of filler words. Though CARE guidelines have been introduced as the most specialized reporting instrument of such studies since 2013, only 6 journals in our dataset (12%) were found to endorse it. The low acceptability of this guideline was confirmed by Agha *et al*. [17] by investigating the JCR-indexed surgical journals. They found only 2 out of 193 journals adhered to the CARE guidelines. Journals with the explicit endorsement of the CARE statement in their instructions to authors present higher completeness of reporting [18].

Such an unwelcome attitude may have arisen because the CARE guide is not adapted to various types of CCRs and is only suitable for traditional CCRs. Moreover, the elements of this guideline have not been accurately explained and elaborated and authors and editors are not proficient in implementing it. Other reasons include the fact that it does not cover the reporting requirements of various areas, and this has led to the development of several extensions [for example, Surgery CAse REport guidelines (SCARE), Homeopathic clinical CAse REport (HOME-CASE), Case Report in Chinese medicine (CARC), and Anaesthesia Case Report (ACRE)] [19–22]. However, the accurate identification of the causes of this issue requires more extensive investigation in perspective of the journals’ editors.

## Conclusions

The scientific publication in the field of medicine is diverse in terms of methodology and study designs due to its historical background and sensitivity concerning human life. International organizations always design and update different standards, criteria, and frameworks to monitor and enhance scientific processes. However, such monitoring has been ignored in the field of CCRs due to challenges, such as the lack of adherence to common scientific structures, the emergence of different formats, and the dominance of population-based approaches instead of personalized medicine. Nevertheless, the electronic publishing environments provided the ground for the publication of a significant volume of such articles in different scientific journals, and the clinical knowledge available in them can be applied by implementing emerging data science technologies (for example, natural language processing, text mining, and knowledge discovery in big data). Improving the quality of the publication, integrating the policies of journals, and updating reporting guidelines of CCRs are some essential steps that should be taken to use such valuable clinical knowledge.

## Supplementary Information

**Additional file 1: Appendix S1.** Description for typology of case reports published in medical journals (*N* = 50).

## Data Availability

All data generated or analyzed during this study are included in this published article (and its Additional file 1: Appendix S1).

## Abbreviations

CARE: CAse REport guidelines
SCARE: Surgery CAse REport guidelines
HOME-CASE: Homeopathic clinical CAse REport
CCRs: Clinical Case Reports
CPD: Continuing Professional Development
