# Supplementary material for: The reporting requirements of case reports and adherence of case report reporting guidelines in medical journals: an analysis of the authors’ guide sections
Source: J Med Case Rep. 2023 Jan 5;17:2. doi: 10.1186/s13256-022-03710-2 (PMC9817370; doi:10.1186/s13256-022-03710-2)
Supplement: Supplementary file 1 — Additional file 1: Appendix S1. Description for typology of case reports published in medical journals (N = 50). [file 13256_2022_3710_MOESM1_ESM.docx]

# Appendix S1. descirption for typology of case reports published in medical journals (N = 50)

| **category** | **Sub-category** | **Variant labels/names** | **N of journals** | **Description** |
| --- | --- | --- | --- | --- |
| **Case report** | Traditional Case Report | Case Report, Brief Observation, Concise Report, Brief Clinical Studies, Clinical and Laboratory Observation, Syndrome in Question, Newly Recognized Syndrome, Case Reports in Diverse Populations | 36 | Classical and common structure of case reports (In the form of introduction, case presentation, discussion) is formed in this type of articles. The clinical case may be presented a new syndrome, observation of a specific clinical condition, laboratory or clinical findings, etc. |
|  | Case-based review | -- | Child S Nervous System | The purpose of these type of articles is to review a specific clinical phenomenon or condition and how to manage this phenomenon in a clinical case or sample. Structurally, they are no different from Classical case reports and include introduction, case description, diagnosis, management, prognosis, outcome, and discussion. |
|  | Clinical / Technical note | -- | Pediatrics International;  Neurology | These articles describe a technique or report a problem or skill in the treatment procedure with an emphasis on a clinical case. |
| **Case series** | -- | Case series, short series, | 11 | This type of Observational studies describes or reports three to ten clinical cases that have similar scenarios or findings (encounter, treatment, and diagnosis). Observational aspect means that there is no control group in the study. Structurally, they are either published in IMRaD structure or classical case reports. |
| **Brief report** | -- | -- | 5 | The structure of this type of article is IMRAD. The number of cases in these articles depends on the type of journal as a case report or a cases series. In the methodology section, the authors are required to report the methods used in patient management and the medical indicators measured.  The main differences between these articles and other case reports are in addition to following the IMRAD structure, this is where other observational studies such as original research, laboratory evaluations (such as genetic analysis) are also published as brief reports. In other words, brief reports are not limited to clinical cases. |
| **Video** | -- | E-videos  Operative Nuances  Video in …  Computer Graphics | 5 | The studied clinical phenomenon is prepared in the form of a video. In this regard, authors must submit a separate textual description of the video. For this type of case report, there is a limited need for titles, images, explanations (or summaries) and resources. |
| **Letter to editor** | -- | Case letter  Correspondence  Brief letter  New-data letter | 18 | Case reports can be written in the form of letter to the editor, writing requirements in this type of articles is a case description, discussion and finally two pictures or tables up to 700 words without abstracts, and keywords. Often these types of articles are not indexed in citation databases. |
| **Round** | -- | -- | International Journal Of Dermatology;  Urology | These types of articles are often written by a group of experts and it is possible to publish this type in the form of a case report and a letter to the editor. |
| **Brief /short communication** | -- | -- | Child S Nervous System;  Journal Of Clinical And Diagnostic Research | In addition to the various animal studies, small studies, case studies, and pilot studies that are published under the name "Brief / short communication", case reports and case series are also published in some journals with this label. The structure of these case reports is no different from traditional case reports. |
| **Clinical problem solving** | -- | -- | New England Journal Of Medicine | In these articles, the authors describe the clinical phenomenon step by step at each stage and present their clinical answers and arguments about that phenomenon. |
| **CPD** | Clinicopathological Cases, Therapeutic Vignette, Genetic Report, A Memorable Patient, Patient Viewpoint | -- | Clinical And Experimental Dermatology | This type of article aims to educate fellowships and novice physicians to improve diagnostic skills and teach treatment techniques and approaches, disease treatment, and genetic reporting. In the format of these articles, two multiple-choice questions are considered to examine the reader’s knowledge of the topic discussed in the article.. |
| **Case conferences** | -- | -- | Journal Of Cardiothoracic And Vascular Anesthesia | This type of article has three main parts: case description, discussion and commentary. The first two parts are written by authors from an institute and present a clinical case. In the commentaries part, experts' opinions on the clinical case are collected from different institutions and departments. The information of these experts in addition to the original authors should be provided. Also, it is recommened to use images and references that enhance the educational value of the case. |
| **Clinical image** | -- | Image in …, Image for …, Neuroimage, Photo vignette, Interesting images, At the focal point , Photo essay , Pictures in …  Clinical pictures , Visual diagnosis | 25 | Attractive images (CTs, MRIs, polysomnographic tracings, or setups, etc) of rare and unusual cases with a brief description of the clinical characteristics. no need for abstracts. it included Maximum three images or tables. there are limitations of word, author and references and finally, the quality of the images is important. |
| **Diagnostic dilemmas** | -- | -- | Journal Of Cardiothoracic And Vascular Anesthesia | This article type is divided into two parts: In the first part, a short case with a difficult diagnosis is presented, which can be diagnosed from graphic evidence. In this part, the reader is encouraged to present his possible and experimental diagnoses and . In the second part, the results of further investigation and evaluations that have solved the puzzle or difficult diagnostic situation are presented. High quality figures and/or video clips are vital for these submissions. |
| **Quiz** | challenge | Clinical images challenge (Image quiz), E-challenge, Clinical Challenge in .. , Photo challenge, Clinical challenge | 6 | A scenario of a disease or clinical condition is presented with clinical pictures and then a clinical question is expressed with four phrase options. The questions in this section are usually about diagnosis, specific clinical features, or follow-up. |
|  | Practical teaching case | -- | Gastroenterology | The requirements for in this structure are that first a description of the patient is presented on one page and then, multiple choice questions (usually 4 options) are posed and in the next page the correct answer is described. |
|  | Clinical quiz | Clinical quiz, Photo quiz, Quiz cases, QUIZ your knowledge!”, What is your diagnosis?, How to do it? | 9 | A common mode in quiz-based case reports is that a brief description of a particular clinical phenomenon or finding is provided, along with related images, and then a question is asked about the diagnosis or clinical practice. Finally, the correct answer is presented in the form of a discussion.  In this type of articles, there is no need for abstracts and tables and the questions are expressed in several options. The number of authors is less than other case reports and usually should not exceed three authors. |
|  | Brief case | -- | Journal Of Clinical Microbiology | These clinical cases are submitted to the journal as two separate article: in the first article, after a summary of the clinical case and related discussion; Three or four self-assessment questions are provided. These articles may contain images, tables, and maximum six references. the second part of the Brief Case (“Closing the Brief Case”) will include the answers to the self-assessment questions, with a brief explanation provided for each answer, followed by three to five bulleted, brief take-home points. |
